# Cardiac vagal control and theoretical models of co-occurring depression and anxiety: A cross-sectional psychophysiological study of community elderly

**DOI:** 10.1186/1471-244X-12-93

**Published:** 2012-07-30

**Authors:** Hsi-Chung Chen, Cheryl C H Yang, Terry B J Kuo, Tung-Ping Su, Pesus Chou

**Affiliations:** 1 Department of Psychiatry and Center of Sleep Disorders, National Taiwan University Hospital, Taipei, Taiwan; 2 Community Medicine Research Center & Institute of Public Health, National Yang-Ming University, Shih-Pai, Taipei, 112, Taiwan; 3 Institution of Brain Science and Sleep Research Center, National Yang-Ming University, Taipei, Taiwan; 4 Division of Psychiatry, National Yang-Ming University, Taipei, Taiwan; 5 Department of Psychiatry, Taipei Veterans General Hospital, Taipei, Taiwan

**Keywords:** Cardiac vagal control, Co-occurring depression and anxiety, Heart rate variability, The Hospital Anxiety and Depression Scale, Tripartite model

## Abstract

**Background:**

In order to elucidate the complex relationship between co-occurring depression and anxiety with cardiac autonomic function in the elderly, this study examined the correlation between cardiac vagal control (CVC) and pre-defined, theoretical factors from the Hospital Anxiety and Depression Scale (HADS).

**Methods:**

Three hundred fifty-four randomly selected Chinese male subjects aged ≥65 years and living in the community were enrolled. CVC was measured using a frequency-domain index of heart rate variability.

**Results:**

Confirmatory factor analysis showed that the flat tripartite model of HADS provided a modest advantage in model fit when compared with other theoretical factor solutions. In the flat tripartite model, there was a significant negative association between anhedonic depression and CVC. In contrast, autonomic anxiety showed a significant positive correlation with CVC. In the hierarchical tripartite model, negative affectivity was not directly associated with CVC; instead, it had positive and negative indirect effects on CVC via autonomic anxiety and anhedonic depression, respectively. As scores for negative affectivity increased, these specific indirect effects diminished.

**Conclusions:**

Among competing models of co-occurring depression and anxiety, constructs from tripartite models demonstrate fair conformity with the data but unique and distinct correlations with CVC. Negative affectivity may determine the relationship of anhedonic depression and autonomic anxiety with CVC. Separating affective symptoms under the constructs of the tripartite models helps disentangle complex associations between co-occurring depression and anxiety with CVC.

## Background

It is well known that depression influences the development of cardiovascular disease in several ways [1-4]. However, several features characterize the association between depression and cardiovascular events. First, there are gender differences in cardiovascular disease and comorbid depression [5]. Second, the differential magnitude of association has prompted researchers to identify high-risk groups with clusters of cardiac-noxious depressive symptoms [6-9]. Third, anxiety symptoms, which commonly co-occurred with depression, have also been linked to adverse cardiac outcomes and are thought to significantly confound the effect of depression on cardiovascular disease [10-12]. In order to further elucidate the nature of gender differences, heterogeneous relationships, and confounding anxiety symptoms in the association between depression and cardiovascular diseases, it is important to explore the role and relevance of biological substrates that underlie theses patterns.

Cardiac vagal control (CVC) reflects the extent to which the tonic vagal activity influences the heart [13]. High resting CVC and a high capacity for withdrawing CVC during environmental demand are thought to facilitate physical and psychological function. In contrast, low resting CVC and a low capacity for withdrawing CVC are thought to predict poor outcomes. Pathways by which the frontal cortex modulates vagal activity via subcortical inputs have been identified [14]. Therefore, CVC exerts regulatory control over attentional and emotional systems as well as behavioral flexibility [15,16].

Impaired CVC has been suggested to mediate the link between depression and cardiovascular events [17,18]. In parallel, there are also gender differences [19,20] and heterogeneous relationships [21-23] in the associations between depressive symptoms and CVC. Moreover, because depression and anxiety are broad, interrelated, affective constructs, co-occurring anxiety symptoms may confound or exacerbate impaired CVC as well [18,24]. These factors obscure the clinical utility of treating affective symptoms for the prevention and treatment of cardiovascular disease. Therefore, we need gender-specific studies that concurrently deal with the associations between co-occurring affective symptoms and heterogeneous patterns with CVC. As such, pre-defined, theory-based management of psychopathology is crucial for preventing post-hoc arbitrary manipulation of predictive variables [24].

Early research on the structure of depression and anxiety resulted in the two-factor model that emphasized two orthogonal dimensions: negative affect and positive affect. Negative affect is a shared, non-specific component to both depression and anxiety, whereas low positive affectivity is a specific factor primarily related to depression [25]. Clark and Watson (1991) extended the two-factor model to the tripartite model, which includes anxious arousal [26]. In the tripartite model, negative affectivity is a shared characteristic between depression and anxiety. The measure of negative affectivity is strongly related to the trait of neuroticism [27,28]. Negative affectivity can be regarded as the distinctive core emotional process of neuroticism [29]. Hence, it can be expressed as not only worry, nervousness, and tension, but also other negative emotional states, such as guilt, anger, and self-dissatisfaction [30]. Investigators further modified the flat tripartite model to a hierarchical structure in order to explain the co-morbidity and specificity of anxiety and depression at the level of both symptoms and disorders [31,32]. The hierarchical tripartite model posits a higher order negative affect factor and two lower order factors specific to the unique components of depression and anxiety (low positive affect and anxious arousal, respectively) in order to describe the relationship among measures of negative affect [26,33]. In the hierarchical arrangement of the three-factor model, a second-order dimension of negativity was conceptually extracted from corresponding lower order depression and anxiety. Consequently, the traditional syndromes of depression and anxiety represent narrow constructs that are highly interrelated. In contrast, the negative affectivity dimension emerges as a broader, more general construct that represents the strong degree of overlap between the lower order syndromes [34]. This model also takes into account the overlap among neuroticism, anxiety, and depression and is applicable to both clinical and non-clinical samples [35]. Multi-group analyses have suggested that the model can be effectively applied to older populations [36].

The above-mentioned series of *a-priori* defined models (i.e. the two-factor model and flat and hierarchical tripartite models) for co-occurring depression and anxiety have been used to examine the psychometric properties of paper-and-pencil measurements [37-39]. These models simultaneously handle the issues of symptomatic heterogeneity and co-occurring affective symptoms; therefore, with distinct affective constructs, they are also good approaches for tackling the complex relationship between affective symptoms and innate biological factors. In the two-factor model, positive affectivity rather than negative affectivity is strongly influenced by endogenous rhythms [40]. In the tripartite model, low positive affectivity is related to reduced right-hemisphere activity, and high negative affectivity is associated with increased left-hemisphere activity. Specifically, anxious arousal was related to increased right-hemisphere activity [41,42]. In addition to brain activity, the tripartite model has also been used to explore association patterns between individual factors and serum cortisol levels [42,43]. Because factors derived from the aforementioned theoretical-based models activate different brain areas, and chronotropic control of heart rate is right-hemisphere dominant, it is possible that different affective constructs correlate distinctively with CVC. However, this potential unique relationship between affective constructs and CVC has never been examined.

Thus, the present study aimed to disentangle the correlation patterns between co-occurring depression and anxiety with CVC in a gender-specific sample. The conformity of competing theory-based models for co-occurring depression and anxiety in an elderly Chinese male population was examined first. Furthermore, each theoretical model was applied to test and compare their unique correlation with CVC. It is hypothesized that each theory-derived construct behaves differently in terms of their relationship with CVC.

## Method

### Participants

This study was a part of the community health survey conducted by the Community Medicine Research Center at the National Yang-Ming University in Taiwan. The subject sampling method has been reported previously [19]. Briefly, in 2007, 392 elderly men (65 years of age and older) dwelling in an urban community located in northern Taipei were randomly selected to participate in the study. Demographic data and a self-reported medical history with respect to hypertension, diabetes, cardiovascular disease, exercise habits, and substance exposure were collected. Exclusion criteria included: (1) failure to provide a history of medical illnesses, (2) not receiving regular treatment for medical illnesses, (3) subjects with conditions which affected cardiovascular fluctuations (i.e. frequent atrial fibrillation or ventricular contractions), and (4) subjects taking antidepressants. A total of 38 potential subjects were excluded, and data from 354 eligible subjects were analyzed. All participants provided written informed consent, and the Ethics Committee of National Yang-Ming University approved this study.

### Measurement of depressive and anxiety symptoms

The Hospital Anxiety and Depression Scale (HADS) is a valid instrument for screening or measuring both clinical and sub-clinical depression and anxiety in the general population [44]. During its development, the authors excluded symptoms that might arise from somatic aspects of illness, which made it useful in the elderly population. The authenticity of the Mandarin version of HADS has been previously investigated [45].

**Figure 1:**
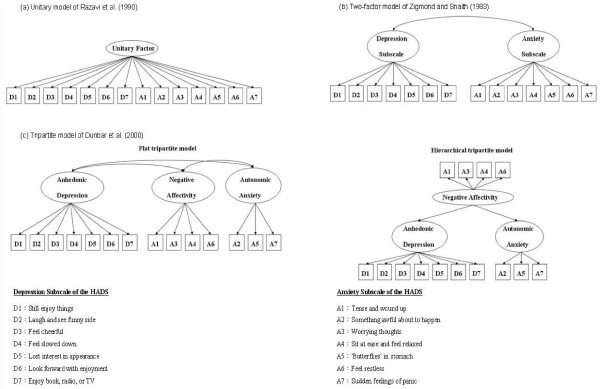
Diagrammatic illustration of the factor structures of the Hospital Anxiety and Depression Scale.

Given the aforementioned evolution of the phenotypic structures of depression and anxiety, HADS had been reported with respect to several theoretical factor solution models. The item distributions of the unitary factor model of Razavi (1990) [46], the original two-factor model of Zigmond and Snaith (1983) [47], and the tripartite three-factor model (flat and hierarchical) of Clark and Watson (1991) [26] are shown in Figure 1. Dunbar et al. (2000) [37] later split the original anxiety subscale of HADS into two factors: negative affectivity and autonomic anxiety. All items on the depression subscale were regarded as good markers for anhedonic depression (Figure 1).

### Measurements of cardiac vagal control

After a 10-min rest in a seated position, a lead-I electrocardigram (ECG) was taken for 5 min. A heart rate variability (HRV) analyzer (SSIC, Enjoy Research Inc., Taiwan) acquired, stored, and processed the ECG signals. Signals were recorded using an 8-bit analog-to-digital converter with a sampling rate of 512 Hz. All peaks of the digitized ECG signals were detected using a spike detection algorithms [48]. The mean and standard deviation of the amplitude and duration of all spikes were used as the standard QRS template. According to the likelihood in fitting the standard QRS template, ventricular premature complex and noise were excluded, and only valid QRS complexes were collected in the procedure of R-R interval rejection. All R-R intervals were used to derive a temporary mean and standard deviation for standard reference. Each R-R interval was then examined. If the standard score of an R-R value exceeded 3, it was regarded as erroneous or nonstationary and was then rejected. Stationary R-R interval values were resampled and interpolated at a rate of 7.11 Hz to produce continuity in the time domain. A nonparametric method of fast Fourier transformation was utilized to perform power spectral analysis. The direct current component was deleted, and a Hamming window was used to attenuate the leakage effect. The power spectrum of high frequency (HF: 0.15-0.40 Hz) was defined as the measurement of CVC. The spectral power of HF was logarithmically transformed to correct the skewed distribution.

### Data analysis

The Statistical Package for the Social Science (SPSS) version 8.0 and the Linear Structural Relations (LISREL) version 8.2 were used to analyze all data. The factor structure of HADS was determined using Confirmatory factor analysis (CFA) with the SIMPLIS language in the LISREL system. The weighted least-square estimator was used to evaluate model fit as this method of estimation can be used reliably with ordered categorical level data and dependably with modest samples sizes. No model revision was made for poorly fitting solutions. In the hierarchical tripartite model, the covariance of first-order factors of anhedonic depression and autonomic anxiety was constrained to be zero.

Multiple goodness-of-fit tests were used to evaluate the models. A root mean square error of approximation (RMSEA) with values <0.08 indicated a good fit, while values >0.10 suggested an unsatisfactory fit. Comparative Fit Index (CFI) >0.90 indicated a good fit to the data, while the Consistent Akaike information criterion (CAIC), Akaike information criterion (AIC), and expected cross validation index (ECVI) allowed for comparison between models [37]. These parameters comprise the three major categories of goodness-of-fit tests: absolute index (RMSEA, ECV), incremental index (CFI), and parsimony index (AIA, CAIC). Thus, this combination examines model fitness more comprehensively.

In multivariate analyses, multiple linear regressions were performed to analyze the relationship between factor solutions of HADS and CVC. Age, education, body mass index, smoking status, weekly frequency of exercise, sites of data collection, and history of hypertension, diabetes, and coronary heart disease were forced into the models to adjust for potential confounding effects. Because of the reported non-linear relationship between biological markers and depression [19,42,43] and the relatively low symptom severity of depression in the present study, the depression subscale of HADS was transformed to ordinal scores based on the quartile distribution and the suggested cut-off point for depression subscale of HADS. The optimal cut-off point for the depression subscales of HADS in community-dwelling Chinese elderly is 6 [49]; therefore, the lowest two quartiles (<5) were grouped as the first class, and the third quartile (5–6) and forth quartile (≥7) were re-classified as 2 and 3, respectively.

Compared with the flat tripartite model, the hierarchical arrangement offers additional information about how each construct influences CVC. The *a-priori* defined hierarchical tripartite model implied that the higher order construct of negative affectivity might confer indirect effects on CVC by the following pathway: negative affectivity → anhedonic depression → CVC and negative affectivity → autonomic anxiety → CVC. In the mediation models, the total effect of negative affectivity on CVC can be further partitioned into a direct effect and two specific indirect effects. A direct effect is interpreted as the effect of negative affectivity on CVC that is independent of the pathway through intervening variables (i.e. anhedonic depression and autonomic anxiety). The indirect effect is the amount by which two cases who differ by one unit on negative affectivity are expected to differ in CVC through negative affectivity’s effect on anhedonic depression or autonomic anxiety, which in turn affects CVC [50]. Indirect effects were statistically examined with the SPSS macro provided by Preacher and Haynes (2008) for assessing indirect effects in multiple mediator models [51]. This macro utilizes a bootstrapping strategy to test the validity of indirect effects. Several modern approaches are used to inference mediation effect. The conventional approach of Baron and Kenny infers the existence rather than quantifies the mediation effect [52]. Another popular inferential technique is the product of coefficients approach (i.e. Sobel test) [53,54]. The ratio of the path coefficients to its standard error is the test statistic for examining the null hypothesis with the *p*-value derived from the standard normal distribution. However, the Sobel test requires not only that the paths that constitute the indirect effects follow a multivariate normal distribution, but also that the sampling distribution of the total and specific indirect effects are normal [51]. In contrast, the bootstrapping strategy quantifies the indirect effect and makes no assumption of multivariate normal distribution in the sampling of indirect effects [50]. Simulation research also shows that bootstrapping is one of the more valid and powerful methods for testing the effects of intervening variables [55,56]. In short, current evidence suggests that the bootstrapping methods are superior to methods that assume symmetry or normality of the sampling distribution of the indirect effect. In the present study, the indirect effects of negative affectivity on CVC were bootstrapped with 5,000 samples; the bias-corrected and accelerated 95% confidence interval (BCa 95% CI) was estimated. Finally, since a higher order construct (i.e. negative affectivity) may moderate the effect of a lower level construct on CVC (i.e. anhedonic depression and autonomic depression), another SPSS macro provided by Preacher et al. (2007) was utilized to examine the conditional indirect effect (i.e. moderated mediation effect) [57].

## Results

### Participants’ characteristics

Table 1 summarizes the demographic and clinical characteristics of participants. There were a total of 354 subjects (mean age: 77.9 ± 5.2 years), and 69.2% of the data was collected at the participants’ homes. Older elderly individuals (≥75 years of age) accounted for 77.9% of the subjects. The mean total score of HADS was 7.0 ± 4.7. The median HADS subscale scores were 4 for depressive symptoms (range: 0–17) and 2 for anxiety symptoms (range: 0–14).

**Table 1:** Demographic and clinical characteristics of participants (n = 354)

	**(n, %)**
Age (years)	
65-74	78 (22.0 %)
75-79	163 (46.0 %)
≧80	113 (31.9 %)
Education (years)	
< 7	100 (28.2 %)
7-9	69 (19.5 %)
10-12	80 (22.6 %)
≧13	105 (29.7 %)
Body mass index (kg/m^2^)	
≧ 25	139 (39.3 %)
Weekly frequency of exercise	
≧ 1/week	292 (82.5 %)
Smoking status	
Current smoker	47 (13.3 %)
Sites of data collection	
Home	245 (69.2 %)
Hospital	109 (30.8 %)
Medical history	
Diabetes mellitus (n, %)	75 (21.2 %)
Hypertension (n, %)	217 (61.3 %)
Coronary heart disease (n, %)	141 (39.8 %)
Hospital Anxiety and Depression Scale	
Total score (mean ± SD)	7.0 ±4.7
Depression subscore (mean ± SD)	4.8 ± 3.2
Quartile 1 (0–2)	78 (22.0 %)
Quartile 2 (3–4)	131 (37.0 %)
Quartile 3 (5–6)	60 (16.9 %)
Quartile 4 (≧7)	85 (24.0 %)
Anxiety subscore (mean ± SD)	2.2 ± 2.1
Quartile 1 (0)	87 (24.6 %)
Quartile 2 (1)	74 (20.9 %)
Quartile 3 (2–3)	113 (31.9 %)
Quartile 4 (≧4)	80 (22.6 %)

### The CFA for various factor solutions of HADS

The factor models and accompanying fit indices are shown in Table 2. Among competing models, the unitary factor model of Razavi (1990) had the worst fit for the data. Except in RMSEA, the flat tripartite model of Dunbar et al. (2000) showed a modest advantage over the hierarchical tripartite model and the original two-factor model. In contrast, the two-factor model of Zigmond and Snaith (1983) provided a comparable fit to the hierarchical tripartite model across all model fit indices.

**Table 2:** Factor structure of the Hospital Anxiety and Depression Scale as determined by testing the fit of models derived from factor analysis

**Models**	**WLS *X***^**2 **^**(d.f.)**	**RMSEA**	**CFI**	**CAIC**	**AIC**	**ECVI**
Unitary factor model of Razavi et al.(1990)	369.37 (77)	0.10	0.86	561.71	425.37	1.21
Two-factor model of Zigmond and Snaith (1983)	297.08 (76)	**0.09**	0.89	496.29	355.08	1.00
Tripartite model of Dunbar et al.(2000)						
Flat tripartite model	283.05 (74)	**0.09**	**0.90**	**496.00**	**345.05**	**0.98**
Hierarchical tripartite model	295.68 (75)	**0.09**	0.89	501.76	355.68	1.01

Bold indicates best model fit as a function of model fit index criteria. Abbreviations: Weighted least-square (*WLS*), Root mean squared error of approximation (*RMSEA*), Comparative fit index(*CFI*), Consistent Akaike information criterion (*CAIC*), Akaike information criterion (*AIC*) and expected cross validation index (*ECVI*).

### Multiple linear regression analyses for the association between factor solutions and CVC

Table 3 shows the relationship of various factor solutions of HADS with CVC. The bivariate correlation between depression subscores of HADS and CVC was examined first. The original depression subscores were not correlated with CVC (Pearson correlation coefficient: *r =* −0.05, *p =* 0.33). In contrast, the transformed ordinal subscale of depression showed a significant negative correlation with CVC (Pearson correlation coefficient: *r =* −0.15, *p =* 0.01). Therefore, the rescaled depression subscale was adopted in models II to IV. The factor derived from the unitary factor model of Razavi (1991) (model I) did not correlate with CVC. Throughout models II to IV, anhedonic depression (rescaled depression subscale) invariably showed a negative association with CVC. In model III, when the tripartite factors of Dunbar et al. (2000) were entered into the model, negative affectivity had no direct effect on CVC [β (SE) = −0.09 (0.05), *p =* 0.12]; in contrast, autonomic anxiety showed a positive correlation with CVC [β (SE) = 0.24 (0.09), *p =* 0.01]. In model IV, the total effect (direct plus indirect effect) of negative affectivity on CVC was not statistically significant [β (SE) = −0.06 (0.04), *p =* 0.17]. Furthermore, the indirect effects on CVC conferred by negative affectivity through anhedonic depression and autonomic anxiety were examined simultaneously in a multiple mediator model. The BCa 95% CI showed that negative affectivity had significant but opposite indirect effects on CVC, either via anhedonic depression [β (SE) = −0.05 (0.03), 95% CI: -0.11, -0.01] or via autonomic anxiety [β (SE) = 0.08 (0.03), 95% CI: 0.02, 0.15].

**Table 3:** **Multiple regression analysis on the association of cardiac vagal control and the Hospital Anxiety and Depression Scale**^**†**^

	**High frequency component of heart rate variability [ln(ms**^**2**^**)]**	
**Factor models**	**β (SE)**	**P-value**
**Model I. Unitary factor model of Razavi (1999)**		
Total score of full scale	−0.01 (0.01)	0.46
**Model II . Original two-factor model of Zigmond & Snaith (1983)**		
Anhedonic depression^‡^	−0.21 (0.09)	0.02
Anxiety subscale	0.02 (0.04)	0.54
**Model III. Flat tripartite model of Dunbar et al. (2000)**		
Anhedonic depression	−0.22 (0.09)	0.01
Negative affectivity	−0.09 (0.05)	0.12
Autonomic anxiety	0.24 (0.09)	0.01
**Model IV. Hierarchical tripartite model of Dunbar et al. (2000)**		
**Direct effect**		
Anhedonic depression	−0.22 (0.09)	0.01
Negative affectivity	−0.09 (0.05)	0.12
Autonomic anxiety	0.24 (0.09)	0.01
**Indirect effect**	β (SE)	BCa 95% CI^§^
Negative affectivity → Anhedonic depression	−0.05 (0.03)	(−0.11, -0.01)
Negative affectivity → Autonomic anxiety	0.08 (0.03)	(0.02, 0.15)

†Variables forced to enter into the regression models include: Age (years), Education (≧13, 10–12, 7–9, <7 years), Diabetes mellitus (yes/no), Hypertension (yes/no), Cardiovascular disease (yes/no), Frequency of exercise (≧ 1 vs <1/week), Current smoker (yes/no) , Site of data collection (Hospital vs home), Body mass index (≧ 25 , vs <25 kg/m^2^), and various combination of factor items.

‡Total score of depression subscale was rescaled by quartiles as <5 (lowest 2 quartiles), 5–6 and ≧7.

§BCa: bias corrected and accelerated confidence interval derived from 5000 bootstrap samples.

**Figure 2:**
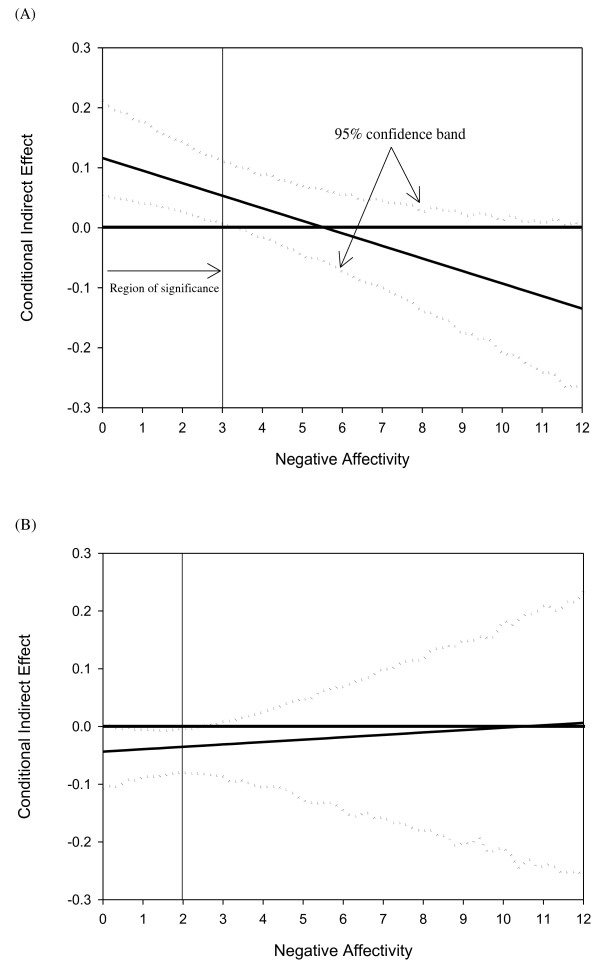
**Plots of specific indirect effect on cardiac vagal control versus the moderator (negative affectivity).** (**A**) conditional indirect effect mediated by autonomic anxiety (**B**) conditional indirect effect mediated by anhedonic depression.

Figure 2 depicts how negative affectivity, under the hierarchical tripartite model, exerted its indirect effect on CVC. Panel A shows that the indirect effect of negative affectivity conferred by autonomic anxiety was conditioned (moderated) on negative affectivity itself. In general, increasing scores of negative affectivity tended to reverse this specific indirect effect from positive to negative. As such, higher negative affectivity reversed the relationship between autonomic anxiety and CVC. Once scores of negative affectivity reached ≥ 4, the specific indirect effect (i.e. negative affectivity → autonomic anxiety → CVC) became statistically non-significant. In parallel, panel B illustrates that the significant indirect effect of negative affectivity on CVC conferred by anhedonic depression (i.e. negative affectivity → anhedonic depression → CVC) was conditioned on scores of negative affectivity ranging from 0 to 3. Therefore, as scores of negative affectivity reached 4 or more, the direct effects of anhedonic depression [β (SE) = −0.16 (0.37), *p =* 0.67] and autonomic anxiety [β (SE) = −0.03 (0.30), *p =* 0.91] on CVC became non-significant but negative coefficients. The indirect effects that negative affectivity conferred through anhedonic depression and autonomic anxiety vanished as well, but the total effect of negative affectivity on CVC became statistically significant [β (SE) = −0.41 (0.19), *p =* 0.04].

## Discussion

In the present study, a series of *a priori*-defined, theoretical factor solution of HADS were used to examine their psychometric conformity and psychophysiological relationship with CVC in elderly males residing in the community. Our results indicated that the flat three-factor model of HADS performed better in fitting the data but provided only a limited advantage over the hierarchical three-factor model and the original two-factor model. However, factors derived from the tripartite model offered an opportunity to scrutinize the counterbalancing effects of anhedonic depression and autonomic anxiety on CVC. In the hierarchical tripartite model, with lower negative affectivity, anhedonic depression and autonomic anxiety exerted counterbalancing effects on CVC along with corresponding indirect effects conferred by negative affectivity on CVC. In contrast, higher negative affectivity alone may reflect the overall deleterious effect of co-occurring anxiety and depression on CVC. Negative affectivity may be the unifying and potentially deleterious element linking individual trait negative emotions to impaired CVC [58]. The present study further illustrated how negative affectivity moderated the relationship between negative emotions and CVC.

Theory-derived factor solutions have been previously utilized to investigate complex relationships between physiological indicators and co-occurring depression and anxiety. For example, the three hypothesized symptom groups in the tripartite model reflect highly distinctive patterns of brain activity [59-61]. CVC is another commonly applied psychophysiological indicator. The CVC has been of significant value in illuminating the process of basic dimensions of psychopathology and in predicting adverse health outcomes [62]. To the best of our knowledge, this is the first study that applies a series of *a priori*-defined, theory-based factor solutions to examine the relationship between CVC and co-occurring affective symptoms. Interestingly, our results suggest that clusters of affective symptoms derived from tripartite models as well as brain activity reflect distinctive patterns of CVC.

In terms of CVC, the results here seem to offer an advantage in utilizing the tri-dimensional scoring of HADS. This helps to disentangle the complex relationship between psychopathology and physiologic markers when applying the flat tripartite model in studies that explore the associations among CVC and co-occurring depression and anxiety. For example, by extracting symptoms of autonomic anxiety from the original anxiety subscale, we demonstrated that the absence of a correlation between the original HADS anxiety subscale and CVC resulted from the heterogeneous relationships between anxiety symptoms and CVC. From the perspective of the hierarchical arrangement of the three factors of the tripartite model, additional information is acquired as primacy is conferred on the factor of negative affectivity. In the present study, negative affectivity not only indirectly affected CVC via autonomic anxiety but also moderated the direct effect of autonomic affectivity on CVC. In other words, the higher order construct of affective symptoms may possess a determinant role in the way that lower order affective constructs affect CVC and eventually lead to adverse health outcomes.

According to the conceptual organization of the tripartite model, negative affectivity explains the covariation between depression and anxiety; however, depression and anxiety have distinct features not shared with each other or with negative affectivity [26]. With respect to cardiac autonomic control, the distinctive relationships between each tripartite factor of HADS and CVC, as noted in the present study, support this presumption. Paradoxically, the positive association between autonomic anxiety and CVC in the preset study subverts the typical understanding that elevated anxiety accompanies sympathetic activation and parasympathetic withdrawal. Several explanations help support this interesting finding.

First, the issues of construct and linguistic validity of autonomic anxiety in HADS may lead to the misallocation of symptom items and cause a biased association between autonomic anxiety and CVC. From the viewpoint of construct validity, Caci et al. (2003) argued that items assigned by Dunbar et al. (2000) were not suitable markers for the constructs of negative affectivity and autonomic anxiety. Furthermore, they were regarded as measuring the same thing [63]. However, in both the Chinese and non-Chinese literature examining the psychometric properties of HADS, the three items selected as markers of autonomic anxiety by Dunbar et al. (2000) nearly matched some forms of anxiety constructs, such as generalized anxiety, psychic anxiety, or panic [64]. Therefore, issues of item misallocation and language translation are insufficient to explain the positive correlation between autonomic anxiety and CVC as noted in this study. Despite arguments about inadequate psychometrical validity, the present study suggests that autonomic anxiety and negative affectivity as defined by Dunbar et al. (2000) seem to be different things in psychophysiological terms.

Another possible explanation is that Ahern et al. (2001) observed that inactivation of the right hemisphere by intracarotid sodium amobarbital administration resulted in a significant decrease in HRV. They suggested that the right hemisphere had a greater role in regulating cardiac function, perhaps by modifying parasympathetic effects [65]. Since autonomic anxiety is associated with the activation of the right parietal region [60], it is reasonable to infer that the positive association between autonomic anxiety and right hemisphere activation may contribute to increased CVC.

In the analysis of conditional indirect effect, we found that the positive contribution to CVC from autonomic anxiety was present only when scores of negative affectivity were low. The association tended to be reversed when negative affectivity scores were higher. Because negative affectivity in the tripartite model refers to “temperamental sensitivity to negative stimuli” and is related to neuroticism, low negative affectivity may represent less emotional arousal and a better coping style during stress. Constructive coping is related to better vagal tone [66]. Hence, in subjects with low negative affectivity, autonomic anxiety may merely be an external marker for good psychological flexibility in response to stress, which is associated with high vagal tone [67]. Several mild physical stresses have been reported to counteract the negative effects of aging and to increase longevity [68]. Herrmann et al. (2000) also reported the protective effect reflected in the HADS anxiety subscale among subjects who received routine exercise testing [69]. When subjects have high negative affectivity (a score of 4 for negative affectivity in this study), the combined effect on CVC from all factors of the tripartite model remains detrimental to health.

There are some limitations to the present study. First, although the flat tripartite model showed a better fit than did the hierarchical tripartite model and the original two-factor model, the difference was modest. Moreover, the goodness-of-fit indices of RMSEA and CFI suggested that none of these competitive models had a reasonably good fit. Empirically, the tripartite model of the European version of HADS provided a better and more acceptable fit to the data than did the two-factor model. In contrast, in the current study sample of elderly Chinese males, some elements may have affected the conformity of the tripartite model to the data. Structural differentiation of self-reported depression and anxiety is less easily performed in an older community sample than in younger populations [70]. In a Chinese population with coronary heart disease, Wang et al. (2006) also found structural ambiguity in the Chinese version of HADS [39]. They suggested that before any clear advantage of the tripartite models over the bi-dimensional models could be demonstrated, the amount of time consumed and the lack of a comparison to related literature rendered the tri-dimensional scoring approaches of HADS highly premature [39]. However, from the standpoint of CVC, the advantages of the analytic strategy of the tri-dimensional approach of HADS override the shortcoming of structural ambiguity. Because the present study did not aim to offer factor structures that conformed to the data with the most optimal goodness-of-fit, we preserved the original model specifications proposed by Dunbar et al. (2000); therefore, no model revisions were performed. Potential sources of specification errors are number of factors, indicators, and error theory. Further studies with larger sample sizes and indicators for latent variables should help to examine the conformity of the tripartite model among elderly Chinese. However, the development of psychometric measurements is usually driven by philology. Perhaps the validation of paper-and-pencil tests with biological markers, as in the present study, maximizes the practical utility of psychometric tools and goes beyond philology.

Second, the study subjects came from an urban community. Their scores for depression and anxiety were too low to generalize the study finding to a general clinical population. Subjects with higher negative affectivity were so few that the statistical power to detect a conditional indirect effect was compromised. However, the total effect of negative affectivity on CVC remained statistically significant when it was 4 or more. As such, the effect was strong enough to be detected even in a small sample. Nonetheless, further research with study subjects that include clinical patients are still necessary in order to determine whether negative affectivity actually moderates the association of autonomic anxiety with CVC.

Third, gender differences are noted in the link between depression and cardiovascular disease. The association between poorer CVC and depressive symptoms has been selectively observed in elderly males only of Chinese ethnicity [19]. However, other studies have demonstrated a female predominance in the link between depression and cardiovascular disease [5]. With respect to gender differences, whether or not different pathophysiological processes exist in different ethnic groups is unclear.

Finally, the confounding effect of medications could not be totally controlled in the present study. Because most classes of antidepressant have robust suppressive effects on CVC [71-75], subjects taking antidepressant were excluded in the present study. In addition to antidepressants, there are other medications with definite or probable effects on CVC, such as beta-blockers [76] and antipsychotics [77]. Unfortunately, we did not collect information on these medications, and future studies must assess these confounds.

## Conclusions

In a comprehensive review, Rottenberg (2007) suggested the necessity of evaluating the effects of depression and anxiety on CVC, both independently and jointly [24]. The psychometric advantage of HADS echoes the requisites that Rottenberg (2007) called for. In the present study, individual associations between CVC and factors of theory-based models of HADS have been delineated among elderly males in the community. The composite scores of HADS have been dissected according to the tripartite model, and this provides the optimal application of HADS in identifying cardio-noxious affective symptoms. The significant and unique effects of negative affectivity and autonomic anxiety on CVC provide a more careful inspection of the way that anxiety symptoms confound the association between depression and CVC, not only in a co-occurring pattern but also in a heterogeneous form. The extent to which this strategy accurately predicts health outcomes remains unknown. Longitudinal studies are necessary to determine the clinical use of tri-dimensional HADS scoring, which is validated by virtue of CVC.

## Competing interests

The authors declare that they have no competing interests.

## Authors' contributions

HCC and PC analyzed the data and drafted the manuscript. CCHY and TBJK helped with the analysis and interpretation of HRV. TPS and PC participated in the study design and coordinated the conduct of the study. All authors read and approved the final manuscript.

## Pre-publication history

The pre-publication history for this paper can be accessed here:

http://www.biomedcentral.com/1471-244X/12/93/prepub
